# The Moral Side

**Published:** 1892-11

**Authors:** 


### The Moral Side.

In an address before the Marion-Sims College (*Alienist and Neurologist*, July, 1892) Dr. C. H. Hughes says:

The study of the physician includes the moral as well as the physical well-being of man, for the purity of the soul has much to do with the health of the body. The purity of the heart and the dominance of the body by principles of rectitude has much to do with the health and consequent happiness of present and succeeding generations. The direct and hereditarily entailed diseases which are the offspring of sin, and *vice versa*, which have filled and are filling the land with misery and woe, both physician and divine are alike especially interested in preventing. The psychology of sin and the pathology of crime are studies alike for doctor and divine.

The man who is sick in his soul is seldom well in his body, and the soul's affairs do not prosper well when the body is disordered.

Like the divine, the physician may also aid in healing "wounded in spirit and the broken hearted," and in "binding up their wounds." He may "minister to a mind diseased," and "with sweet oblivion's antidote cleanse the stuffed bosom of that perilous stuff which weighs upon the heart." He does this effectually through the modern successful management of melancholia.—*Med. and Surg. Reporter.*

